# Sex specificity of resistance to caTAUstrophe

**DOI:** 10.1002/alz.71716

**Published:** 2026-08-06

**Authors:** Maria Carrigan, Colin Birkenbihl, Hannah M. Klinger, Oliver Langford, Gillian T. Coughlan, Mabel Seto, Jane A. Brown, Annie Li, Madison Cuppels, Michael Properzi, Jasmeer Chhatwal, Julie Price, Aaron Schultz, Dorene Rentz, Rebecca Amariglio, Harm J. Krugers, Rik Ossenkoppele, Keith Johnson, Reisa Sperling, Timothy J. Hohman, Michael Donohue, Rachel F. Buckley

**Affiliations:** ^1^ Alzheimer Center Neurology Vrije Universiteit Amsterdam The Netherlands; ^2^ Amsterdam Neuroscience Neurodegeneration Amsterdam The Netherlands; ^3^ Faculty of Science Swammerdam Institute for Life Sciences University of Amsterdam Amsterdam The Netherlands; ^4^ Massachusetts General Hospital Harvard Medical School Boston Massachusetts USA; ^5^ Alzheimer's Therapeutic Research Institute University of Southern California San Diego California USA; ^6^ Brigham and Women's Hospital Harvard Medical School Boston Massachusetts USA; ^7^ Vanderbilt Memory and Alzheimer's Center Vanderbilt University School of Medicine Nashville Tennessee USA

**Keywords:** Alzheimer's disease, global cognition, inverse learning approach, magnetic resonance imaging (MRI), neocortical tau resistance

## Abstract

**INTRODUCTION:**

As amyloid beta (Aβ) accumulates, tau pathology spreads beyond medial temporal lobe (MTL) into neocortical (NEO) regions, though some older adults resist this progression, or what we call here “caTAUstrophe.” Given previous evidence of higher tau levels in women, we tested how tau resistance presented in men and women separately.

**METHODS:**

Employing data from 872 Aβ+ older adults across three cohorts, we trained sex‐specific penalized linear regression models in individuals experiencing caTAUstrophe (females: N_Train_ = 172; males: N_Train_ = 121) to predict the expected NEO tau levels. We estimate resistance as lower‐than‐expected NEO tau levels in training‐independent individuals (N_Test_ = 579) to assess sex‐specific resistance associates.

**RESULTS:**

Relative feature importance in sex‐specific expectation models differed in 97.7% of variables (false discovery rate‐adjusted *p* value < 0.001). Age and Aβ burden associated with male resistance, while Clinical Dementia Rating, latent Preclinical Alzheimer's Cognitive Composite, and adjusted hippocampal volume were associates in both sexes.

**DISCUSSION:**

Our study highlights sex‐specific biological and clinical factors in the prediction of NEO tau and associates of resistance. Understanding sex‐specific resistance pathways informs targeted Alzheimer's interventions.

## Highlights

- We estimate sex‐specific NEO tau resistance in Aβ‐positive adults.
- Relative feature importance differs markedly between male‐ and female‐only models.
- Higher age and lower amyloid burden uniquely associated with resistance in men.
- Higher cognition and greater hippocampal volume associated with resistance in both sexes.

## 1 BACKGROUND

Under the ATN framework,^1^ individuals who are positive for both amyloid beta (Aβ+) and tau (T+) pathology meet the criteria for Alzheimer's disease (AD) classification, irrespective of cognitive status. Despite the required presence of both pathologies, neocortical (NEO) tau density in particular is linked most strongly to cognitive decline.^2^, ^3^ Early tau accumulation in the medial temporal lobe (MTL), in particular the entorhinal cortex, is an early precursor to rapid tau proliferation into the neocortex in preclinical AD.^4^ The transition from MTL to NEO tau is not inevitable; while the presence of Aβ and higher MTL tau burden accelerates this “caTAUstrophe”,^4^, ^5^ considerable heterogeneity exists in the extent to which an Aβ+ individual might progress to NEO T+.^6^ This variability raises an important question: Why do some Aβ+ individuals, who may also possess other risk factors including older age, apolipoprotein E (*APOE*) ε4 carriership, or female sex, remain low in NEO tau burden (i.e., reflecting NEO tau resistance), while others predictably follow this path? Female sex in particular presents a compelling factor potentially modulating resistance to NEO tau burden, given well‐established sex differences showing higher MTL and NEO tau levels in women,^7^ faster rates of tau accumulation and proliferation,^8^ and reduced overall brain resilience to tau pathology^9^ compared to men. Therefore, the current study aimed to investigate whether and how resistance to NEO tau burden presented differently in men and women. Among Aβ+ older adults from three cohorts, we separately estimated male and female NEO tau resistance using the recently published inverse learning approach.^10^ Sex‐specific models were built to estimate NEO tau resistance. We first examined how resistance presented in women and men separately (i.e., “what is resistance”) by looking at our expectation models themselves. Subsequently, using our models to estimate resistance in women and men, we tested which factors associated with NEO tau resistance in each sex.

## 2 METHODS

### 2.1 Sample characteristics

A total of *N* = 872 Aβ+ individuals across the AD continuum were selected from the Anti‐Amyloid Treatment in Asymptomatic Alzheimer's Disease trial and the companion Longitudinal Evaluation of Amyloid Risk and Neurodegeneration study^11^ (A4/LEARN, *N* = 388), the Harvard Aging Brain Study^12^ (HABS, *N* = 72), and Alzheimer's Disease Neuroimaging Initiative^13^ (ADNI, *N* = 412). Across all cohorts, we included participants with demographic data, including age, sex, education as measured in years, diagnosis, race/ethnicity classified as (self‐reported) Hispanic/Latino versus non‐Hispanic/Latino, *APOE* ε4, as well as longitudinal cognitive performance and longitudinal magnetic resonance imaging (MRI). We conducted this study under the ethical guidelines stipulated by the Partners Human Research Committee, which is the Institutional Review Board for the Massachusetts General Hospital and Brigham and Women's Hospital. The study was carried out in accordance with the guidelines of the Declaration of Helsinki. Written informed consent was obtained from all participants.

### 2.2 Imaging measures

MRI: Three‐dimensional (3D) structural T1‐weighted MRI scans were acquired across all cohorts using previously detailed site‐specific scanners.^14^, ^15^, ^16^ T1 images were segmented and parcellated using FreeSurfer software (version 6.0). Hippocampal volume (HV) and total gray matter volume were examined after adjusting for intracranial volume (ICV). MRI scans were selected based on closest date to each participant's tau positron emission tomography (PET) scan.

PET: Tracers for Aβ PET acquisition were 18F‐Florbetapir (FBP) in ADNI and A4/LEARN, 18F‐Florbetaben (FBB) in ADNI, and 11C Pittsburgh compound‐B (PiB) in HABS. The PET acquisition parameters for each study are described elsewhere.^12^, ^17^, ^18^, ^19^ Distribution volume ratios (DVRs) were calculated using Logan plotting between 40 and 60 min after injection for HABS and between 50 and 70 min after injection for ADNI and A4/LEARN to develop standardized uptake value ratios (SUVRs). For each cohort, summary measures were derived from a weighted average within a large aggregate cortical region of interest (ROI) that included the precuneus, rostral anterior cingulate, medial orbitofrontal, superior frontal, rostral middle frontal, inferior parietal, inferior temporal, and middle temporal regions (collectively referred to as FLR). The target composite was referenced to cerebellar gray for HABS, and the whole cerebellum for ADNI and A4/LEARN (FBP and FBB tracers). In ADNI, FBP cortical summary SUVRs were obtained from data previously processed by the University of California Berkeley, accessed through the LONI data portal (http://adni.loni.usc.edu/). In brief, FBP SUVRs were computed by averaging retention values across cortical ROIs from the lateral and medial frontal, anterior and posterior cingulate, lateral parietal, and lateral temporal regions, with reference to the whole cerebellum. As different tracers and processing pipelines were used across cohorts, Aβ‐PET signal was dichotomized (positive/negative) using published criteria: 1.11 (FBP) and 1.08 (FBB) SUVR threshold in ADNI,^13^ 1.15 SUVR threshold and visual read in A4/LEARN,^20^ and a 1.185 DVR threshold in HABS.^21^

For tau PET, 18F‐Flortaucipir (FTP) was used in all three cohorts, with study‐specific FTP‐PET acquisition parameters published previously.^22^, ^23^, ^24^ FTP‐PET images were generated based on mean retention between 75 and 105 min (ADNI), 90 and 110 min (A4/LEARN) and between 80 to 110 min (HABS) after injection. FTP‐PET data were processed using in‐house pipelines,^22^ using cerebellar gray as reference region for calculation of SUVRs. Cross‐sectional tau‐PET composites were calculated for NEO, composed of bilateral fusiform, middle temporal, inferior parietal, and inferior temporal regions, and MTL, composed of bilateral amygdala, parahippocampal, and entorhinal regions. Processing of T1‐weighted images was performed with FreeSurfer version 6.0 to identify gray‐white and pial surfaces, as well as to produce automatic Desikan‐Killiany cortical and aseg subcortical ROI parcellations.^25^ All quality control measures were carried out as previously described.^12^

### 2.3 Cognitive outcome measures

We used the global score of the Clinical Dementia Rating (CDR)^26^ and the Preclinical Alzheimer's Cognitive Composite score (PACC)^27^ for our analyses. Due to different variants of the PACC that are used in each cohort,^28^ we calculated the latent PACC (lPACC) to harmonize this cognitive composite measure across cohorts. Harmonizing cognitive scores circumvents the drawbacks of standardizing, which imposes strong, and often violated, assumptions on the psychometric properties of the neuropsychological tests.^28^

### 2.4 APOE genotyping

Genotyping of *APOE* was performed in each study through blood sample collection. *APOE* ε4 positivity was determined as heterozygous and homozygous carriers of the ε4 allele (one or two copies, respectively), excluding those carrying the ε2/ε4 combination.

### 2.5 Inverse learning model

We employed the recently published inverse learning approach^9^ to operationalize NEO tau resistance as a continuous metric. Until now, the field has relied heavily on the standard residual approach to quantify the concept of (resilience and) resistance; however, growing concerns have highlighted that this method is suboptimal and statistically inappropriate in certain circumstances.^28^, ^29^ We have shown that the standard residual approach makes certain implicit (and often uncontrollable) assumptions, which may lead to biased or incorrect resistance estimates, for instance, an unknown proportion of resistant participants in the training data, ignoring multicollinearity, and the low explanatory power of the outcome (e.g., predicting NEO tau).^9^ Thus, neglecting these statistical assumptions can lead to residual estimates that are dominated by model error with little or no information left of the concept of interest, in this case, resistance. The inverse learning approach was designed to address and overcome these limitations by building the residual estimating models only on data with low probability of containing resistant participants. Including resistant participants in your training models runs the risk of learning the variance of interest and leaving only model error in the residuals.

**RESEARCH IN CONTEXT**

1. **Systematic review**: We reviewed studies on tau spread, amyloid pathology, and resistance in AD, focusing on sex differences. Prior work characterized NEO tau accumulation and its cognitive consequences, but mechanisms underlying resistance to tau spread, and how they differ between men and women, remain underexplored. Relevant studies on predictors of tau pathology, cognition, hippocampal integrity, and amyloid burden were included.
2. **Interpretation**: Our findings reveal sex‐specific differences in associates of NEO tau resistance. Cognition and hippocampal volume associated with resistance in both sexes, whereas age and amyloid burden were uniquely associated with resistance in men. Relative feature importance differs markedly in male‐ versus female‐only models, highlighting divergent pathways of resistance.
3. **Future directions**: This work provides a framework for studying sex‐specific resistance to tau spread. Future studies could explore molecular and lifestyle factors contributing to resistance, validate predictive models in larger cohorts, and develop targeted, sex‐informed interventions to slow AD progression.

Briefly, the inverse learning approach entails training a model to predict the “expected” outcome (i.e., the expected level of NEO tau) based on a sample that fulfills the criteria of low resistance or “typical” pathological progression (i.e., individuals with high levels of both amyloid and NEO tau). We call this predictive model the expectation model. The inverse learning approach publication demonstrated that including all predictors into the expectation model increases its predictive accuracy because it learns better how to predict the expected outcome, which serves as the reference point to which the residual is computed, thereby leading to more accurate estimates. The trained expectation model is then applied to a heterogeneous and independent sample. Resistance to NEO tau is then calculated by subtracting the empirically observed values from the predicted expected values. Resistance is thereby operationalized as the deviation from the predicted expectation, where a negative residual indicates lower tau resistance while a positive residual indicates greater tau resistance. In addition, we have developed an error correction model that additionally corrects for the expectation model's error based on the training phase,^9^ thereby further reducing the bias created from model error.

### 2.6 Operationalization of (sex‐specific) resistance

**FIGURE 1:**
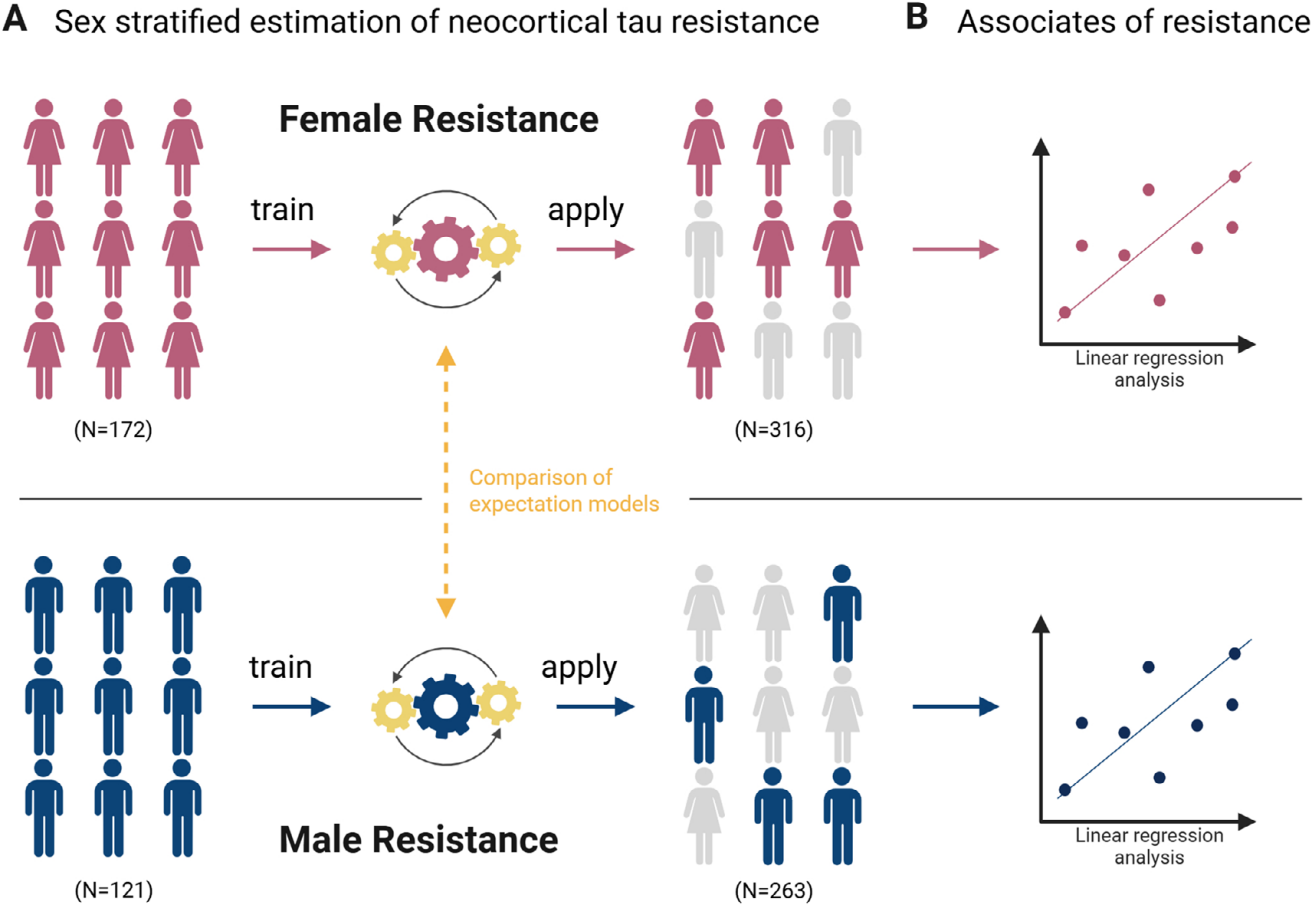
Study overview. Operationalization of sex‐specific resistance to neocortical tau. (A) Training two separate expectation models in male (N_Train _= 121) and female populations (N_Train _= 172) that experienced a more typical clinical progression during their lives and subsequent application of each trained model on a heterogeneous population (N_Test _= 579) stratified by men (*N* = 263) and women (*N* = 316) to estimate resistance and comparison of expectation models in terms of model feature importance (highlighted in yellow). (B) Investigating associates of male and female resistance to neocortical tau within each model using linear regression analyses. Created with BioRender.com.

In our study, the training sample was defined a priori following a presumed disease progression according to the ATN framework.^1^ As such, we only included individuals experiencing caTAUstrophe: those who were Aβ+ and NEO T+ at baseline or started without abnormal pathology but converted at some point throughout the study to Aβ+ and T+. We included every visit of the selected participants for model training (including any Aβ− and T− visits) to ensure that the model learned to predict expected tau levels across disease stages. Tau positivity was defined using a Gaussian mixture model, as explained in the Supplementary Material. We were specifically interested in how the expectation of resistance was estimated in each sex separately and whether different associates of resistance became apparent in each sex. Thus, our initial training sample (*N* = 293) was stratified by sex (*N*_males _= 121, *N*_females _= 172), resulting in two separate samples used to train respective expectation models predicting NEO tau burden, one in each sex (male‐only/female‐only; Figure 1A). We used the same range of predictors for both models (153 features in total, including demographics such as age, sex, education, race/ethnicity, known risk factors such as *APOE* ε4, cognition, MTL tau SUVR, ICV‐adjusted hippocampal volume, continuous Aβ burden, and metabolic/lifestyle factors such as body mass index, smoking, and blood pressure). The models used to predict NEO tau were penalized linear regression models (elastic net). Due to their ridge regression and LASSO regression penalty terms, they are suited for large predictor spaces and are robust against predictor collinearity. Demographic information on the male and female training data (expectation sample) is provided in Tables S1 and S2. The data used for training the sex‐stratified models were not used in any of the subsequent analyses. We estimated sex‐specific resistance to NEO tau by applying the female‐only expectation model to women (*N* = 316) and the male‐only expectation model to men (*N* = 263) separately (Figure 1A). Demographic information for the analyzed participants is provided in Tables 1 (women) and 2 (men). All subsequent linear regression analyses investigating the association of NEO tau resistance with our variables of interest were performed on female resistance as estimated in women using the female‐only model and male resistance as estimated in men employing the male‐only model, respectively (Figure 1B). For the purpose of this study, we will refer to “female‐only” and “male‐only” as the expectation samples that were used to build the models predicting NEO tau burden in each sex, while “women” and “men” will refer to the heterogenous sample of potentially resistant individuals that each sex‐specific model was applied to for estimation of female and male resistance, respectively.

**TABLE 1:** Female sample demographics.

Characteristic	Overall *N* = 3161	A4/LEARN *N* = 1711	ADNI *N* = 1141	HABS *N* = 311
Age	73 (7) [57, 94]	72 (5) [65, 86]	74 (8) [57, 94]	78 (7) [66, 92]
Years of education	15.81 (2.62) [9.00, 22.00]	15.56 (2.75) [9.00, 22.00]	16.20 (2.35) [12.00, 20.00]	15.81 (2.77) [11.00, 20.00]
Diagnosis				
Cognitively normal	280 (89%)	171 (100%)	81 (71%)	28 (90%)
MCI	31 (9.8%)	0 (0%)	28 (25%)	3 (9.7%)
Dementia	5 (1.6%)	0 (0%)	5 (4.4%)	0 (0%)
Race/ethnicity				
Hispanic or Latino	15 (4.7%)	5 (2.9%)	9 (7.9%)	1 (3.2%)
Not Hispanic or Latino	296 (94%)	162 (95%)	104 (91%)	30 (97%)
Unknown	5 (1.6%)	4 (2.3%)	1 (0.9%)	0 (0%)
APOE ε4 (ε4+)	167 (55%)	95 (56%)	51 (50%)	21 (70%)
Unknown	14	0	13	1
Baseline MTL tau	1.18 (0.13) [0.87, 1.62]	1.17 (0.12) [0.87, 1.58]	1.19 (0.14) [0.90, 1.62]	1.24 (0.13) [1.06, 1.52]
Baseline neocortical tau	1.17 (0.08) [0.93, 1.35]	1.16 (0.08) [0.93, 1.35]	1.20 (0.07) [1.01, 1.35]	1.19 (0.07) [1.06, 1.35]
Baseline amyloid	56 (28) [6, 160]	56 (28) [6, 160]	53 (28) [18, 137]	62 (29) [24, 126]
Unknown	6	3	2	1
MMSE	28.82 (1.53) [14.00, 30.00]	28.88 (1.17) [24.00, 30.00]	28.62 (2.07) [14.00, 30.00]	29.16 (1.07) [27.00, 30.00]
Unknown	12	0	12	0
lPACC	0.64 (0.48) [−1.72, 2.00]	0.71 (0.37) [−0.07, 2.00]	0.46 (0.57) [−1.72, 1.72]	0.89 (0.49) [−0.12, 1.90]
Unknown	13	0	12	1
Resistance	−0.25 (0.33) [−1.29, 0.66]	−0.25 (0.33) [−1.29, 0.59]	−0.28 (0.34) [−1.23, 0.66]	−0.13 (0.31) [−0.67, 0.41]

Abbreviations: A4/Learn, Anti‐Amyloid Treatment in Asymptomatic Alzheimer's Disease trial and the companion Longitudinal Evaluation of Amyloid Risk and Neurodegeneration; ADNI, Alzheimer's Disease Neuroimaging Initiative; APOE ε4, apolipoprotein E ε4 allele; HABS, Harvard Aging Brain Study; lPACC, latent Preclinical Alzheimer's Cognitive Composite; MCI, mild cognitive impairment; MMSE, Mini‐Mental State Examination; MTL, medial temporal lobe.

^1^Mean (SD) [Min, Max]; *n* (%)

**TABLE 2:** Male sample demographics.

Characteristic	Overall *N* = 2631	A4/LEARN *N* = 1301	ADNI *N* = 1061	HABS *N* = 271
Age	76 (7) [60, 95]	73 (5) [65, 86]	78 (7) [60, 95]	78 (6) [68, 88]
Years of education	16.79 (2.81) [8.00, 30.00]	16.89 (2.97) [8.00, 30.00]	16.75 (2.58) [10.00, 20.00]	16.44 (2.95) [12.00, 20.00]
Diagnosis				
Cognitively normal	202 (77%)	130 (100%)	45 (42%)	27 (100%)
MCI	44 (6.5%)	0 (0%)	44 (42%)	0 (0%)
Dementia	17 (6.5%)	0 (0%)	17 (16%)	0 (0%)
Race/ethnicity				
Hispanic or Latino	5 (1.9%)	2 (1.5%)	3 (2.8%)	0 (0%)
Not Hispanic or Latino	256 (97%)	126 (97%)	103 (97%)	27 (100%)
Unknown	2 (0.8%)	2 (1.5%)	0 (0%)	0 (0%)
APOE ε4 (ε4+)	133 (51%)	65 (50%)	56 (53%)	12 (46%)
Unknown	2	0	1	1
Baseline MTL tau	1.19 (0.12) [0.91, 1.55]	1.17 (0.12) [0.93, 1.47]	1.22 (0.13) [0.94, 1.55]	1.18 (0.09) [0.91, 1.32]
Baseline neocortical tau	1.16 (0.08) [0.94, 1.34]	1.14 (0.07) [0.94, 1.33]	1.19 (0.08) [0.99, 1.34]	1.14 (0.07) [0.96, 1.25]
Baseline amyloid	62 (32) [0, 193]	59 (30) [0, 193]	65 (35) [22, 152]	69 (28) [33, 128]
Unknown	11	1	4	6
MMSE	28.21 (1.85) [20.00, 30.00]	28.38 (1.43) [25.00, 30.00]	27.76 (2.32) [20.00, 30.00]	29.15 (1.06) [27.00, 30.00]
Unknown	3	0	3	0
lPACC	0.40 (0.53) [−1.57, 1.70]	0.51 (0.32) [−0.30, 1.47]	0.15 (0.64) [−1.57, 1.62]	0.81 (0.42) [0.23, 1.70]
Unknown	4	0	3	1
Resistance	−0.30 (0.46) [−2.50, 0.89]	−0.32 (0.49) [‐2.50, 0.89]	−0.33 (0.40) [−1.32, 0.49]	−0.07 (0.42) [−0.95, 0.68]

Abbreviations: A4/Learn, Anti‐Amyloid Treatment in Asymptomatic Alzheimer's Disease trial and the companion Longitudinal Evaluation of Amyloid Risk and Neurodegeneration; ADNI, Alzheimer's Disease Neuroimaging Initiative; APOE ε4, apolipoprotein E ε4 allele; HABS, Harvard Aging Brain Study; lPACC, latent Preclinical Alzheimer's Cognitive Composite; MCI, mild cognitive impairment; MMSE, Mini‐Mental State Examination; MTL, medial temporal lobe.

^1^Mean (SD). [Min, Max]; *n* (%)

### 2.7 Statistical analyses

Statistical analysis was performed in R version 4.3.2 (R Foundation, Vienna, Austria). We performed quality control on all demographic and other variables (e.g., cognition, metabolic, lifestyle) to ensure direct translation across cohorts. Because our sex‐specific models are fundamental to deriving our resistance estimates, we investigated the feature importance of the same predictors in the female‐only and male‐only expectation models to assess how our models used to estimate resistance differed across sexes as a result of their biological and clinical characteristics. For this, we estimated the difference in means for the same variable's coefficients between the sex‐specifc models as 95% confidence intervals calculated across 1000 bootstrap samples. Feature importance was quantified using the variables’ coefficients in the model. Additionally, we performed sensitivity analyses by statistically matching our expectation samples on variables that significantly differed between the sexes, in our case age and lPACC, to reduce known sex biases that exist in these variables (Supplementary Material, Tables M1‐2 and Figures M1‐7). We next investigated potential associates (age, cognition, education, MTL tau, ICV‐adjusted hippocampal volume, Aβ burden, and MTL tau × Aβ) of tau resistance within each sex. For this, we ran a series of linear regression models in women using resistance scores estimated by our female‐only expectation model and, in a separate series, in men using resistance scores estimated by our male‐only expectation model. In each of these analyses, potential associates were standardized before fitting the model and covariates were age, education, *APOE* ε4 status, and cohort.

## 3 RESULTS

### 3.1 Sex‐specific model feature importance

**FIGURE 2:**
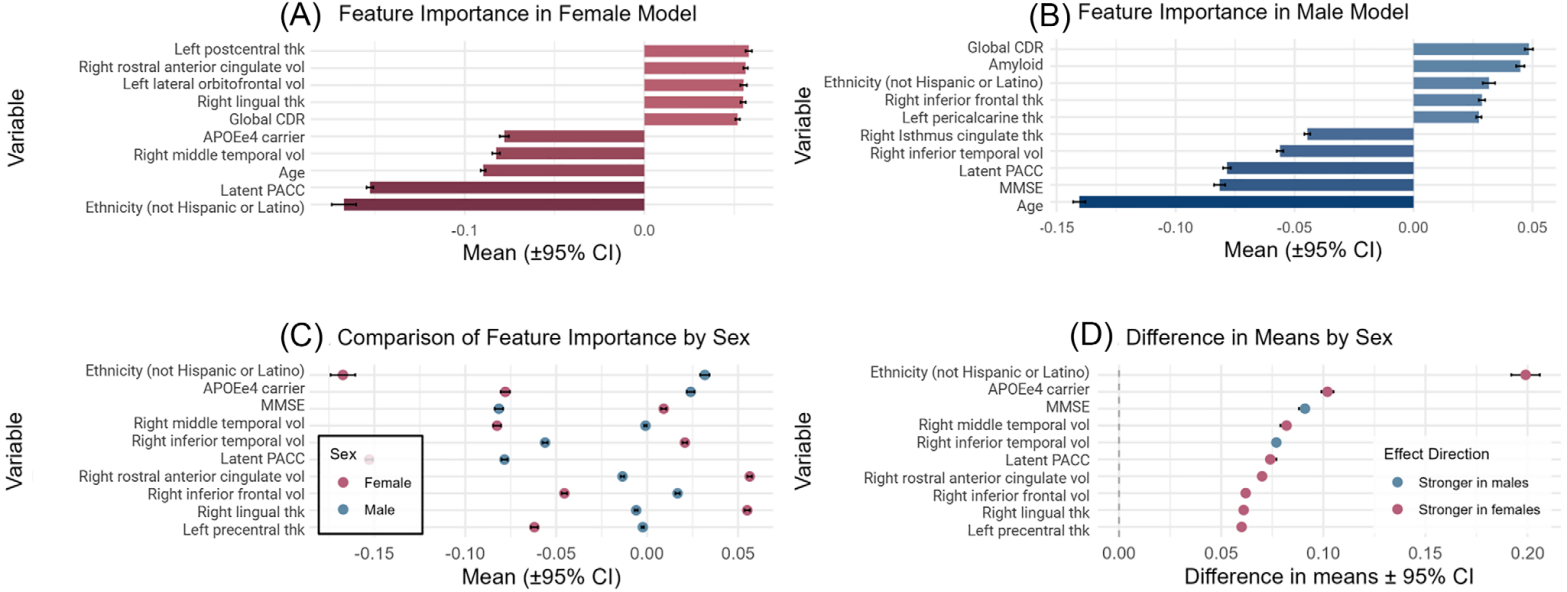
Sex differences in feature importance for sex‐specific expectation models. Top 10 mean bootstrapped estimates of feature importance (± 95% confidence interval, top 5 positive and top 5 negative) in sex‐stratified expectation models are shown separately for females (A) and males (B), ordered by descending mean importance of features. Direct comparison of top 10 mean bootstrapped feature importance (± 95% confidence interval) from male and female models plotted jointly for each feature for visual comparison of sex‐specific model contributions (C). Difference in means between female and male feature weights of top 10 (highest magnitude) variables (± 95% confidence interval) are shown in (D), reflecting the relative strength and direction of sex effects on model feature importance. Values in blue indicate greater importance in the male model, while pink values indicate greater importance in the female model.

When assessing the concordance of the most important (top five positive and top five negative; Figures 2A,B) features in the female‐only and male‐only models, we found that only five of them overlapped (see also Figures S1 and S2 for all features). Cognition, as measured using the global CDR score and lPACC, as well as age and race/ethnicity, were represented as important model features in both models (Figures 2C and Figure S3). Interestingly, the race/ethnicity variable impacted the sex‐specific models in opposite directions. Among those top 10, features distinctively important to the male‐only model included Mini‐Mental State Examination (MMSE) and Aβ, whereas *APOE* ε4 contributed considerably to the female‐only model. Comparing the mean bootstrapped feature importance across the female‐only and male‐only model revealed significant differences for 148 out of the total 153 variables, resulting in a difference in relative feature importance in 97.7% of variables (140 of which are MRI‐related; Figure 2D and Figure S4; see Table S3). Of note, our sensitivity analyses in age‐ and cognition‐matched samples revealed a similar pattern, with a difference in relative feature importance for 92.2% of the variables, suggesting that these differences extend beyond sex‐specific baseline characteristics. Specifically, our main analyses showed that for the sex‐specific prediction of NEO tau levels in our samples, race/ethnicity (Importance in male‐only model, I_m _= 0.03, Importance in female‐only model, I_f _= −0.17, Difference in means, D*_M _* = 0.20, CI [0.19, 0.21]), *APOE* ε4 (I_m _= 0.02, I_f_ = −0.08, D*_M_ *= 0.10, CI [0.05, 0.05]), lPACC (I_m _= −0.08, I_f _= −0.15, D*_M _* = 0.07, CI [0.07, 0.08]), smoking (I_m _= 0.01, I_f _= −0.03, D*_M _* = 0.03, CI [0.03, 0.04]), education (I_m _= −0.00, I_f _= 0.02, D*_M _* = 0.02, CI [0.02, 0.02]), and global CDR (I_m _= 0.05, I_f _= 0.05, D*_M _* = 0.00, CI [0.00, 0.01]) weighted higher in the female‐only compared to the male‐only expectation model. By contrast, MMSE (I_m _= −0.08, I_f _= 0.01, D*_M _* = 0.09, CI [0.09, 0.09]), age, (I_m _= −0.14, I_f _= −0.09, D*_M _* = 0.05, CI [0.05, 0.05]), Aβ (I_m _= 0.05, I_f _= −0.00, D*_M _* = 0.05, CI [0.05, 0.05]), as well as metabolic measures including diastolic and systolic blood pressure (I_m _= 0.01, I_f _= −0.00, D*_M _* = 0.02, CI [0.02, 0.02] and I_m _= −0.01, I_f _= 0.00, D*_M _* = 0.02, CI [0.02, 0.01], respectively), body weight (I_m _= −0.01, I_f _= 0.00, D*_M _* = 0.01, CI [0.01, 0.01]), and pulse (I_m _= 0.01, I_f _= −0.00, D*_M _* = 0.01, CI [0.01, 0.01]) weighted significantly higher in the male‐only expectation model compared to the female‐only expectation model.

### 3.2 Female resistance

**FIGURE 3:**
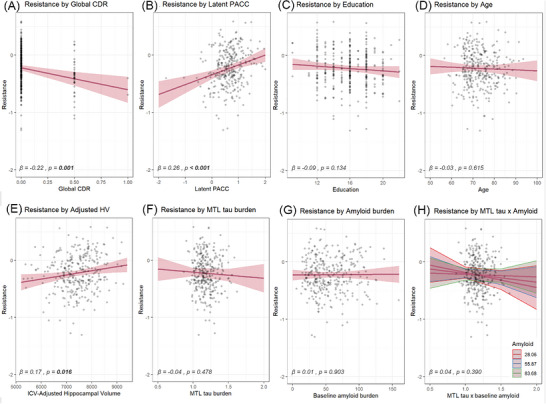
Associates of female resistance to neocortical tau. Higher global CDR associated with lower necortical tau resistance in women (panel A: *β* = −0.22, *p* < 0.01), whereas higher latent PACC was associated with higher female resistance (panel B: *β* = 0.26, *p* < 0.001), as was higher ICV‐adjusted hippocampal volume (panel E: *β* = 0.17, *p* < 0.05). Female resistance was not associated with years of education (panel C: *β* = −0.09, *p* = 0.134), age (panel D: *β* = −0.03, *p* = 0.615), MTL tau (panel F: *β* = −0.04, *p* = 0.476), or amyloid burden (panel G: *β* = 0.01, *p* = 0.903), nor with the interaction of MTL tau and amyloid burden (panel H: *β* = 0.04, *p* = 0.390). Effect sizes are reported as standardized betas (β) to allow comparison across associates.

Higher global CDR scores, reflecting worse cognition, were inversely associated with higher female resistance (Figure 3A; *β* = −0.22, CI [−0.35, −0.09], *p* < 0.01), and higher lPACC, reflecting better cognition, associated with higher female resistance (Figure 3B; *β* = 0.26, CI [0.13, 0.39], *p* < 0.001). Hippocampal volume was significantly associated with higher female resistance (Figure 3E; *β* = 0.17, CI [0.03, 0.30], *p* < 0.05). By contrast, education, age, MTL tau, and Aβ‐PET burden were not associated with female NEO tau resistance (Figures 3C,D,F,G, respectively). Finally, we found no interaction between a MTL tau and Aβ on female NEO tau resistance (Figure 3H).

### 3.3 Male resistance

**FIGURE 4:**
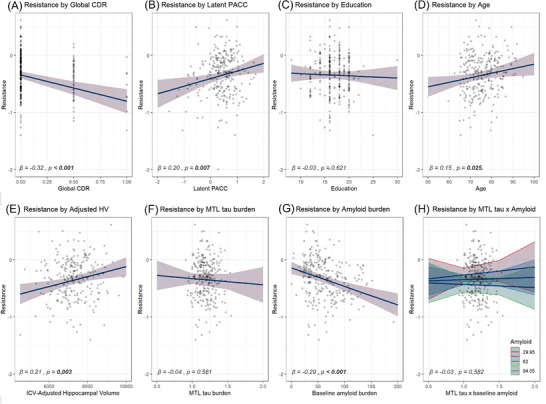
Associates of male resistance to neocortical tau. Higher global CDR (panel A: *β* = −0.32, *p* < 0.001) and baseline amyloid burden (panel G: *β* = −0.29, *p* < 0.001) associated with lower male necortical tau resistance, whereas latent PACC (panel B: *β* = 0.20, *p* < 0.01), age (panel D: *β* = 0.15, *p* < 0.05) and ICV‐adjusted hippocampal volume (panel E: *β* = 0.21, *p* < 0.05) were associates of higher male resistance. In men, resistance was not associated with years of education (panel C: *β* = −0.03, *p* = 0.621), MTL tau (panel F: *β* = −0.04, *p* = 0.561), or the interaction of MTL tau and amyloid burden (panel H: *β* = −0.03, *p* = 0.582). Effect sizes are reported as standardized betas (β) to allow comparison across associates.

Higher global CDR scores inversely associated with higher male resistance (Figure 4A; *β* = −0.32, CI [−0.47, −0.18], *p* < 0.001) and higher lPACC was associated with higher male resistance (Figure 4B; *β* = 0.20, CI [0.05, 0.34], *p* < 0.01). Older age was associated with higher male resistance (Figure 4D; *β* = 0.15, CI [0.02, 0.28], *p* < 0.05), as was ICV‐adjusted hippocampal volume (Figure 4E; *β* = 0.21, CI [0.07, 0.35], *p* < 0.01). By contrast, higher Aβ‐PET burden was inversely associated with higher male tau resistance (Figure 4G; *β* = −0.29, CI [−0.41, −0.16], *p* < 0.001). We found no association between male resistance and education (Figure 4C) or MTL tau (Figure 4F). We found no significant association of a MTL tau and Aβ interaction with male resistance (Figure 4H).

## 4 DISCUSSION

Sex differences in AD pathology have been established extensively across various disease‐related factors, including Aβ‐associated tau burden,^7^ cognitive decline,^29^ and AD progression.^30^ In this study, we employed our recently published inverse learning approach to estimate NEO tau resistance in a sample of female and male individuals who represent the caTAUstrophe, defined as the detrimental spread of tau from MTL to NEO regions.^4^ Specifically, we built two separate sex‐specific models predicting NEO tau burden and applied each model to a sample of women and men, respectively, who potentially included individuals with higher levels of tau resistance. We found that almost all variables differed significantly in their importance for predicting NEO tau between the sex‐specific models. Most notably, cognition (both lPACC and global CDR), education, and *APOE* ε4 showed higher mean feature importance in the female‐only model relative to the male‐only model, whereas age and Aβ showed higher mean feature importance in the male‐only model. Race/ethnicity showed opposite directions in mean feature importance between sex‐specific models, with higher mean feature importance in the female‐only compared to the male‐only model, suggesting an exemplified disadvantage in females who identify as Hispanic/Latino.^31^ Notably, even among cognitive measures, their relative contributions were not uniform across sexes; for instance, MMSE contributed to predicting tau burden in men but not women. This underscores that different commonly used indices of clinical status may capture distinct sex‐specific and sex‐agnostic aspects of tau resistance. Overall, our findings align with well‐established sex differences in AD, particularly with regard to sex differences in susceptibility to tau accumulation and disease progression,^29^, ^30^ highlighting the importance of sex‐related characteristics in each sex when predicting disease progression.

However, it is important to note that in this study, resistance overall was quite low in both men and women, with most individuals’ resistance estimates placing below zero, indicating higher‐than‐expected NEO tau burden relative to model predictions. Interestingly, male resistance in particular presented with a higher skew toward lower resistance. This result persisted in our sensitivity analyses in which we matched female and male expectation samples (Supplementary Material), suggesting that the findings were not attributable to cohort differences in age or disease stage. It is possible that they rather reflect underlying sex‐specific differences in factors contributing to NEO tau resistance. While the mechanisms underlying lower male tau resistance remain to be fully established, one potential explanation relates to sex differences in brain and cognitive reserve. Women have been shown to exhibit relative advantages in cognitive performance,^32^ particularly verbal memory, or greater cortical thickness,^9^ for a given level of AD pathology, suggesting a higher capacity to tolerate or compensate for disease burden. Extending this framework, it is plausible that such reserve mechanisms may also contribute to greater tau resistance in women, whereby NEO tau remains closer to expected levels despite higher overall burden. By contrast, men may exhibit less compensatory buffering, resulting in greater deviation toward higher‐than‐expected tau burden and, thus, lower estimated resistance. These findings, in combination with previous evidence on sex differences in AD‐related pathophysiology, further highlight the need for sex‐specific operationalization of NEO tau resistance.

When investigating sex‐specific associates of resistance in women and men separately, we found that better cognitive performance, as reflected by lower global CDR and higher latent PACC scores, was associated with higher NEO tau resistance in both sexes, indicating that individuals with better cognition were also more resistant to the caTAUstrophe. Our matched analyses further suggest that the association between cognition and resistance in men is attenuated when cognitive performance is matched across groups, indicating that the relationship is contingent on the baseline cognitive differences between men and women in our cohorts. However, years of education – often defined as a proxy measure of cognitive resilience (or better‐than‐expected cognitive performance in the context of AD^33^) – did not associate with resistance in either sex, which is in line with a study by Ramanan and colleagues,^34^ who found no association between education and regional tau burden. Taken together, these findings suggest that baseline cognitive ability or increasing “cognitive buffer” through education might not be the driving factor in higher resistance. Rather, it seems that either some individuals have unique characteristics that are reflected in both less susceptibility to pathology and better cognition or, alternatively, given the downstream effects of tau on cognition,^2^, ^3^ higher resistance is protective of cognitive decline by slowing disease progression. Together, these findings importantly distinguish resistance (avoiding pathology) conceptually from cognitive and brain resilience (coping with pathology), for which education is the most robust determinant.^33^, ^35^, ^36^

In line with previous research, hippocampal volume associated with higher NEO tau resistance in both women and men, indicating a substantial involvement of the hippocampus in brain reserve mechanisms relevant to NEO tau resistance;^37^, ^38^ although our matched analyses suggest that this involvement of the hippocampus might be contingent on the baseline clinical and demographic characteristics that differ between men and women in our cohorts. Interestingly, older age was associated with higher NEO tau resistance in men, suggesting an influence of selective survival, whereby men who survive to older age constitute a subgroup with greater resistance to tau pathology. This aligns well with our matched analyses, as we found no association after this subgroup was removed. In contrast, no comparable association was observed in women, who have a higher tau burden overall. This suggests that as tau spread is already further progressed, age may not explain any additional variance in tau resistance in this group.^8^, ^39^ Concordant with previous findings highlighting different rates of Aβ‐induced acceleration of the caTAUstrophe in women and men, and a female disadvantage in the brain's ability to cope with Aβ proteinopathy,^7^ lower Aβ burden at baseline was associated with higher male resistance but not female resistance. The lack of association between Aβ and resistance in women, contrasting with its strong association in men, suggests that once Aβ is present, NEO tau accumulation in women may proceed more independently of baseline Aβ levels, consistent with prior evidence of accelerated tau propagation in females.^8^ By contrast, Aβ burden remains a key determinant of deviation from expected tau in men, indicating a more tightly coupled Aβ–tau relationship with a more gradual disease progression.^39^ MTL tau burden did not associate with resistance in either sex. Importantly, this should be interpreted in the context of our modeling framework, which was designed to estimate expected NEO tau from non‐tau predictors and therefore does not directly model within‐modality relationships. As such, these findings do not contradict prior evidence that MTL tau is an early and mechanistically important feature of disease progression^4^, ^40^; rather, they suggest that baseline MTL tau alone may not sufficiently capture sex‐specific variability in NEO tau resistance in a cross‐sectional context. In addition, the differential relationship between Aβ burden and sex‐specific resistance was not modulated by MTL tau in either sex, which may also reflect this modeling approach, rather than a lack of biological interaction.

Taken together, the findings of our study highlight existing sex differences in the predictors of NEO tau and associates of resistance. Nonetheless, we acknowledge several limitations. First, estimating NEO tau resistance using our inverse learning approach is influenced by the characteristics of the expectation sample employed for training the predictive model. Running these analyses with a different expectation sample mixture proportion or different measures (e.g., genetic testing for ancestry instead of self‐reported race/ethnicity) could yield differing results. However, we aimed to have a robust expectation sample by pooling several data‐rich cohorts and performing sensitivity analyses using statistically matched expectation samples. Although education did not differ significantly between sexes and was therefore not used as a matching variable in our sensitivity analyses, it remains a potentially relevant factor given the mixed evidence regarding its relationship with tau accumulation.^34^, ^41^ Furthermore, sex stratification of both the expectation sample and our test sample resulted in relatively small sample sizes (which was further reduced in our sensitivity analyses as a result of sample matching), thereby reducing statistical power that potentially limited the identification of relevant associations. Finally, while our study was cross‐sectional, we aim to conduct longitudinal studies of sex differences in NEO tau resistance in future work given its dynamic nature.

In conclusion, we demonstrate the sex‐specific expectation prediction of AD‐related NEO tau burden and the associates of NEO tau resistance. Therefore, we strongly recommend the use of sex‐specific models, as crucial sex‐specific characteristics might counteract one another and lead to a net effect near zero when using pooled‐sex approaches. A better understanding of sex‐specific pathways that promote resistance to NEO tauopathy is necessary to identify protective factors that may be leveraged for targeted interventions.

## CONFLICT OF INTEREST STATEMENT

T.J.H. is on the scientific advisory board of Circular Genomics, serves as deputy editor for *Alzheimer's & Dementia: Translational Research and Clinical Intervention*, and serves as section editor for *Alzheimer's & Dementia*. R.O. is currently a full‐time employee of Eli Lilly and Company. His contribution to the work presented in this manuscript was performed as an employee of Amsterdam University Medical Centers. R.O. has received research funding/support from European Research Council, ZonMw, NWO, National Institutes of Health, Alzheimer's Association, Alzheimer Nederland, Stichting Dioraphte, Cure Alzheimer's Fund, Health Holland, ERA PerMed, Alzheimerfonden, Hjarnfonden, Avid Radiopharmaceuticals, Janssen Research & Development, Roche, Quanterix, and Optina Diagnostics, has given lectures at symposia sponsored by GE Healthcare, received speaker fees from Springer, and was an advisory board/steering committee member for Asceneuron, Biogen, Johnson & Johnson, and Bristol Myers Squibb. All the aforementioned funds were paid to Amsterdam University Medical Centers. All other authors have nothing to disclose. Author disclosures are available in the Supporting Information.

## CONSENT STATEMENT

This study complies with the Declaration of Helsinki. All participants provided written informed consent.

## Supporting information



Supporting Information
